# Rapid development of 56 novel microsatellite markers for the benthic freshwater bug *Aphelocheirus aestivalis* using Illumina paired-end sequencing data and M13-tailed primers

**DOI:** 10.1007/s11033-020-05974-7

**Published:** 2020-11-28

**Authors:** Agnieszka Kaczmarczyk-Ziemba

**Affiliations:** Department of Genetics and Biosystematics, Faculty of Biology, University of Gdansk, Wita Stwosza 59, 80-308 Gdansk, Poland

**Keywords:** Aphelocheiridae, Freshwater true bug, High-throughput sequencing, Microsatellites, Illumina

## Abstract

The freshwater true bug *Aphelocheirus aestivalis* (Aphelocheiridae) is widely distributed in Europe but occurs rather locally and often in isolated populations. Moreover, it is threatened with extinction in parts of its range. Unfortunately, little is known about the genetic diversity and population structure due to the lack of molecular tools for this species. Thus, to overcome the limitations, a whole-genome sequencing has been performed to identify polymorphic microsatellite markers for *A. aestivalis*. The whole-genome sequencing has been performed with the Illumina MiSeq platform. Obtained paired-end reads were processed and overlapped into 2,378,426 sequences, and the subset of 267 sequences containing microsatellite motifs were then used for in silico primer designing. Finally, 56 microsatellite markers were determined and 34 of them were polymorphic. Analyses performed in two samples (collected from Drawa and Gowienica rivers, respectively) showed that the number of alleles per locus ranged from 2 to 21, and the observed and expected heterozygosity varied from 0 to 0.933 and 0.064 to 0.931, respectively. The microsatellite markers developed in the present study provide new suitable tools available for the scientific community to study *A. aestivalis* population dynamics. The assessment of its genetic diversity and population structure will provide important data, that can be used in population management and conservation efforts, elucidating the broad- and fine-scale population genetic structure of *A. aestivalis*.

## Introduction

The benthic freshwater true bug *Aphelocheirus aestivalis* (Fabricius 1794) and two endemic congeneric species from the Iberian Peninsula, *A. murcius* (Nieser and Millán 1989) and *A. occidentalis* (Nieser and Millán 1989) [1] are the only representatives of the family Aphelocheiridae in Europe. *A. aestivalis* is distributed throughout Europe [2], where lives in the middle and upper reaches of streams and rivers with β-mesosaprobic waters [3, 4]. The individuals of *A. aestivalis* occurring in Europe are predominantly micropterous [2]. Due to the fact that only a very few macropterous specimens capable of flight have been collected in the past [5], it has been assumed that *A. aestivalis* disperses mainly through the watercourses [6]. The remarkable downstream drift of nymphs and adults was observed e.g. in the Danube [6] and the Rhein rivers [5].

Although *A. aestivalis* is relatively tolerant of changes in some physical and chemical characteristics of water, it is mainly threatened by industrial and agricultural pollution and the regulation of watercourses [7]. In some European countries, *A. aestivalis* is considered an endangered species and is included in local and national Red Lists or Red Data Books [2]. Successful conservation efforts require not only an understanding of the biology of *A. aestivalis* and its environmental requirements (e.g. [2, 6, 8, 9]) but also the knowledge on the distribution of the genetic diversity in its populations. Therefore, information about the genetic diversity and differentiation should become incorporated into ecological research and further conservation strategies. However, only a few molecular data that analyze the distribution of *A. aestivalis* have been published so far. For instance, fragmentary data have been obtained during a comprehensive study based on DNA barcodes in aquatic Heteroptera [10], the differentiation among related species [3, 11], and in approaches leading to the effectiveness evaluation of new molecular tools [12]. More recently, the variability of selected mitochondrial and nuclear markers was evaluated in five populations of *A. aestivalis* [13]. Analyses revealed low genetic diversity at both levels. Interestingly, endosymbiotic bacteria of *Wolbachia* have been detected in all tested specimens, and its presence could correlate with decreasing mitochondrial diversity of *A. aestivalis*. However, the low genetic variation at both mitochondrial and nuclear level that has been observed in tested samples can also be a result of populations’ close relationships, past demographic phenomena, or a specific feature of *A. aestivalis*.

In the past, microsatellite markers have emerged as one of the most popular and effective molecular tools [14]. Given their codominant and neutral nature, biparental mode of inheritance, and high level of polymorphism, microsatellites have been widely used for determining the genetic divergence among populations, estimation of population structure, and genetic diversity in different insect species (e.g. [15]). Nevertheless, such nuclear markers have not been developed for *A. aestivalis* so far, and thus, the structure of its populations, as well as the genetic divergence among them, have not been determined.

To overcome the limitations described above, a set of novel microsatellite markers has been developed for *A. aestivalis*. Moreover, a fast and cost-effective protocol for species-specific microsatellite markers discovery using Illumina MiSeq sequencing, and the amplification of selected loci with M13-tailed forward primers is presented in this paper.

## Materials and methods

### Sample collection and DNA extraction

Specimens of *A. aestivalis* were collected in 2017 from various rivers located in Central and Western Europe (Table 1). *A. aestivalis* is not an endangered or protected species in the sampling area and sampling activities were not performed at locations where specific permission is required. The samples were collected using dragnet, scraping 0.2 m^2^ of the bottom. The specimens were picked *in situ* from the collected material. All specimens were separately preserved in 70% ethanol and stored at − 20 °C until further analyses.

**Table 1:** Geographic locations of sampling sites, where *A. aestivalis* specimens were collected for described analyses

Sampling site [Country code]	Sample ID	Coordinates
Brda river [PL]	BR	N 53.756944 E 17.761111
Drawa river (near Rzepowo) [PL]	DR	N 53.590231 E 16.096775
Słopica river (outflow of Dominikowo Lake [PL]	DS	N 53.208357 E 15.837871
Gowienica river [PL]	GOW	N 53.680119 E 14.812904
Krąpiel river (inflow to Ina River) [PL]	KI	N 53.318650 E 15.058834
Krąpiel river (near Krzywnica) [PL]	KK	N 53.434298 E 15.192272
Stary potok river [PL]	SP	N 53.309240 E 15.756783
Corgo river [PT]	CR	N 41.380941 W 7.699801

Genomic DNA was extracted from specimens using the High Pure PCR Template Preparation Kit (Roche Diagnostics GmbH, Mannheim, Germany) according to the manufacturer’s protocol. Genetic material was extracted from the abdominal tissue of all collected individuals.

### Microsatellites identification

Total genomic DNA isolated from four individuals collected from different populations (marked as DS, DR, SP, and BR) was pooled and used for library preparation using the TNEBNext® DNA Library Prep Master Mix Set for Illumina® (New England BioLabs Inc.). The sequencing was performed with an Illumina MiSeq sequencer at Genomed (Warsaw, Poland). A 2 × 250 base pair (bp) read mode was used during sequencing. The obtained sequences were trimmed and filtered with the Cutadapt v.1.12 software package [16].

The obtained sequences (2.4 Mega reads) were used for microsatellites detection with QDD v.3.1.2 [17]. Primers were initially designed in the QDD pipeline, but its further analyses (including determining the annealing temperature and the ability to form homo- and heterodimers) were performed with the FastPCR [18] and OligoAnalyzer [19] software.

### Microsatellites amplification and analyses

All forward primers were tagged with M13(-21) (5′-TGTAAAACGACGGCCAGT-3′) tails at the 5′ end [20]. PCR reactions were performed in 20 µL volume containing 0.8x JumpStart *Taq* ReadyMix (1 U JumpStart Taq DNA polymerase, 4 mM Tris-HCl, 20 mM KCl, 0.6 mM MgCl_2_, 0.08 mM dNTP; Sigma-Aldrich, Germany), 0.4 µM of forward and reverse primers, 0.3 µM of fluorescently labeled (6-FAM, HEX or TAMRA) M13 primer and ~100 ng of DNA. The following PCR conditions were used: 1 min initial denaturation at 94°C, followed by 30 cycles of 94 °C for 30 s, gradient temperature for 30 s (range from 52 to 65 °C), and 72 °C for 1 min. Since the M13 primer needs a 53 °C annealing temperature, eight final cycles were added at the end of the PCR cycles to allow the annealing of the M13 primer with the previously formed amplicons (each cycle contained 30 s denaturation at 94 °C, 30 s M13 primer annealing at 53 °C and 1 min elongation at 72 °C). At the end was 7 min final extension at 72 °C. The amplification products were separated by 2% agarose gel electrophoresis in a 1 × SB buffer and visualized with SimplySafe^TM^ (ethidium bromide replacement; EURx, Poland) in UV light.

After the suitable annealing temperatures were determined, the screening for polymorphic and consistently amplifiable loci was performed. In the first step, DNA extracted from 14 *A. aestivalis* individuals collected from seven sampling sites from Central and Western Europe (Poland: specimens from Brda (BR), Słopica (DS), Drawa (DR), Stary Potok (SP) rivers, and two sampling sites from Krąpiel river (KK and KI, respectively), and Portugal: specimens from Corgo river (CR); two individuals per sample;) was used as templates for amplification. Then, microsatellite loci with amplification success were chosen for routine genotyping. Amplification products were run on an ABI Prism 310 DNA Sequencer (Applied Biosystems, Carlsbad, CA, USA) and analyzed with PeakScanner v.2.0 (Applied Biosystems) using GS-500 (ROX) as a size standard. Routine genotyping was performed using template DNA extracted from specimens collected from Drawa (DR; 30 individuals) and Gowienica (GOW; 30 individuals) rivers. Details for developed microsatellite loci are given in Table 2.

**Table 2:** Primer sequences and general description of 56 new microsatellite loci for *Aphelocheirus aestivalis*. 5’ tails attached to forward primers are in brackets

Locus name	Degree of polymorphism	Primer sequence 5′-3′	Repeat motif	T_a_ (°C)	Accession no.
*Aaesti2*	monomorphic	F: [M13]CCGCGCACTTTGTTACACT R:ATTTCGTGGAGTGCAATACG	(ACTGA)_5_	59.9	MT988404
*Aaesti3*	monomorphic	F: [M13]CATTTAATCGTTATTGTAACACGTTC R: AATTGCAAGATTCCACCTGC	(TTTG)_5_	59.9	MT988405
*Aaesti4*	monomorphic	F: [M13]AACAACTTCAAATATAGTTAGGTCGGC R: CCGGTCTCCTTGTACTAATGACT	(ACAA)_5_	51.5	MT988406
*Aaesti5*	polymorphic	F: [M13]TCATTCTGGCTCATTTGCAT R: AAACACTCACATTCAGGAACAGT	(TTTA)_5_	51.5	MT988407
*Aaesti7*	monomorphic	F: [M13]CGATGTGGAGACAGTTTGGA R: GTCATTGGCAGGCTCTTCAT	(TATT)_5_	54.5	MT988408
*Aaesti8*	polymorphic	F: [M13]AGCGCTCAAAGTACTGACAAG R: TGGCGATGTCCTTTATTCCT	(TTAT)_5_	59.9	MT988409
*Aaesti9*	monomorphic	F: [M13]ACTCATAGCCTTTCGTTATCTCTTC R: GAATTTAACTGTAGCCTGTAATTCTGC	(ATTT)_5_	52.9	MT988410
*Aaesti11*	monomorphic	F: [M13]CTGCCATTTGCGTCAACACT R: CCTCCTGCTGCCTTCTCTAA	(TGTC)_5_	52.9	MT988411
*Aaesti12*	polymorphic	F: [M13]GGGAGGGAATCCTTTACAACC R: CAACAATTAAGTAATATTGGACCTCTT	(ACAT)_5_	51.5	MT988412
*Aaesti14*	monomorphic	F: [M13]ATGACGCCCTTCAGGGTAG R: TGTAGGACTACAAGCGACGG	(AGGT)_6_	52.9	MT988413
*Aaesti15*	polymorphic	F: [M13]GGTGAATCTCCGTAAACTTGG R: TTGTAACGACGAAGCACATTG	(ATAG)_5_	51.5	MT988414
*Aaesti16*	polymorphic	F: [M13]AACCTGGGAATTTAATGCAAA R: TGCCCTCAATTGTCTGATTT	(CAA)_6_	54.5	MT988415
*Aaesti17*	monomorphic	F: [M13]GGAATTTGACGATCGTTTCC R: TTTGGTTCCATATGGTTCTCACT	(TTG)_5_	54.5	MT988416
*Aaesti18*	monomorphic	F: [M13]GAGACGGATCCACCATCTGT R: CCATGAATAGTGAGCTCTGGC	(GTT)_5_	54.5	MT988417
*Aaesti19*	polymorphic	F: [M13]TCCTCTGCAGACTCTTTACTGG R: CGGAAGGTGAGGTAGCAGTAG	(CAA)_5_	54.5	MT988418
*Aaesti20*	monomorphic	F: [M13]TGTTGCTATAAACCTGGTAACCC R: GTGGAAGCTCCAGCTTGTTT	(ACA)_5_	54.5	MT988419
*Aaesti21*	polymorphic	F: [M13]TGACACCACCTCCATTGAAA R: TGGGCCCAGGTGATTGAT	(AGT)_5_	54.5	MT988420
*Aaesti22*	polymorphic	F: [M13]ACGCGTACGCAATCAATATCT R: GATTGAGGGAAAGAGAGGCA	(ACT)_5_	54.5	MT988421
*Aaesti23*	monomorphic	F: [M13]AAGATTCAAGAGGCCAAGGG R: GCATGCATCTTTATCACCTGTT	(AGT)_6_	52.9	MT988422
*Aaesti24*	monomorphic	F: [M13]TCTCTCCTTGACTAACGCGG R: AGGTTGGTAACGACAAGGCA	(CTA)_5_	54.5	MT988423
*Aaesti25*	monomorphic	F: [M13]TGGTCAAATATTGTTAAGGAAGCC R: TCGTATCTATGTGAATCGCGTC	(TAG)_6_	51.5	MT988424
*Aaesti26*	polymorphic	F: [M13]AGGAGTGGCAGAAGGATTAGG R: TCCACTCGTCCTCCCTCTAA	(GTA)_5_	54.5	MT988425
*Aaesti28*	polymorphic	F: [M13]GACCCAGAGTGAAAGTGGGA R: GCAAGTGTCAAGGGTTGGTT	(CTA)_5_	54.5	MT988426
*Aaesti29*	monomorphic	F: [M13]CGAAGCAAGGCCTCTGAGTA R: AAGGCCCACTCTGAGCTGTA	(ACT)_5_	54.5	MT988427
*Aaesti30*	monomorphic	F: [M13]GCTACACCTTCGCCATTTGT R: GCTCCTCCACAGTCAGCTTC	(TGG)_5_	54.5	MT988428
*Aaesti31*	monomorphic	F: [M13]CCAGACATTTACACCACTTCCA R: AGAACACATTCCCAGGTTGC	(CAC)_5_	51.5	MT988429
*Aaesti32*	monomorphic	F: [M13]AAATTCCGGTTGTTCTTCCC R: GGGTTGGTTTCAAAGTCACG	(GTG)_5_	54.5	MT988430
*Aaesti33*	monomorphic	F: [M13]GTGTCCGCCTACTGATTGCT R: TCTTTCCGAACAGAACCCAT	(CCA)_5_	51.5	MT988431
*Aaesti34*	monomorphic	F: [M13]ATGATTGAATGGCTCCGGT R: TGGAGGCCCTACATTTCTTG	(ACC)_5_	54.5	MT988432
*Aaesti35*	monomorphic	F: [M13]GGGATTCTATACGCGACTTGC R: ACGGTTATGTTCAAACAGAGGAA	(GTG)_6_	54.5	MT988433
*Aaesti37*	polymorphic	F: [M13]TCCTCTAAAGACGTTGGCGT R: GTTCGACGCACTTCTGGAAT	(GA)_34_	54.5	MT988434
*Aaesti38*	polymorphic	F: [M13]CTTATGTAGCACCACCGAGG R: AGCTGTGATTCTGACCTCTCG	(GA)_29_	54.5	MT988435
*Aaesti39*	polymorphic	F: [M13]AAACGGAGCAGCTCATAACG R: TGGTATAAAGCGCAAGACGC	(GA)_29_	59.9	MT988436
*Aaesti40*	polymorphic	F: [M13]GATTGCGGAGAATGTGGG R: TTATCTTCCGGAGCCCTCTT	(GA)_28_	54.5	MT988437
*Aaesti41*	polymorphic	F: [M13]AAATTCAATGGGAATATTGTTTGA R: TTCATCCTGCAACACCAGTC	(AT)_27_	51.5	MT988438
*Aaesti47*	polymorphic	F: [M13]TCTGCTCCTCAACTCCCTGT R: AAGATTCCGTTTGTTGCGTC	(AT)_23_	50.8	MT988439
*Aaesti48*	polymorphic	F: [M13]TTGAGTAATCAGAGAATAGCAGTCAA R: CCCAATCCACGTAAACATGA	(AT)_23_	50.8	MT988440
*Aaesti49*	polymorphic	F: [M13]TCTCCTTGCTGGTCTTCCC R: CACCTTCCAGAAATCGTTTAAGA	(TA)_23_	50.8	MT988441
*Aaesti50*	polymorphic	F: [M13]AGGAGCCGAAGTGAGAAGAA R: GCTCGCTTTACGTTTGGC	(TA)_23_	50.8	MT988442
*Aaesti51*	polymorphic	F: [M13]TCCGTTCGTTCTTATATTACGC R: GGATTTAATTGGCCAGCTGAT	(AT)_23_	50.8	MT988443
*Aaesti54*	polymorphic	F: [M13]TGGAGAGTGTCACATAAAGTCCC R: GGCCTCCCACCAGCTTAAT	(AT)_23_	50.8	MT988444
*Aaesti55*	polymorphic	F: [M13]AAGCGATGGGAGAATGTTGT R: CGTTAATCCCATCCTCCATT	(CT)_22_	54.5	MT988445
*Aaesti56*	polymorphic	F: [M13]CCTACGTTGGCCCAGTAGAAT R: AGTGAGTCCGTTGCAAGGTT	(GA)_22_	54.5	MT988446
*Aaesti59*	polymorphic	F: [M13]GGGATTTATGGGAGACGGAT R: GCTGGAGCATGTTTCAAGGT	(AT)_22_	54.5	MT988447
*Aaesti60*	monomorphic	F: [M13]CTTATTGGCGTCCTTATGCC R: CGAATTACTCTCATCTTAACAGCTTC	(AT)_22_	50.8	MT988448
*Aaesti62*	polymorphic	F: [M13]CATTCAGACTTGGCATTCTTGA R: TCCCGTGTTAATAACTAACCCATC	(TA)_22_	54.5	MT988449
*Aaesti63*	polymorphic	F: [M13]CGGTCTATATGTCCCTAATGGG R: CAGGCTCCTCAAGTTTCTCAA	(TA)_22_	54.5	MT988450
*Aaesti64*	polymorphic	F: [M13]AAAGGGCTATATTATGAAGTCCCA R: AGTTTGACGACCGAACTCGT	(TA)_22_	54.5	MT988451
*Aaesti65*	polymorphic	F: [M13]CATCACAACACTGGAGGCTG R: TTTAGGGATGAATAAGCTCTGAAAT	(AT)_22_	54.5	MT988452
*Aaesti66*	polymorphic	F: [M13]TGCTGATCAACTTGCCAAAC R: GCGTTTAAGACGAAGAGGGA	(TC)_21_	54.5	MT988453
*Aaesti67*	polymorphic	F: [M13]CAATTCTGGAAACGGAATTAGTG R: AAGAACGACCGTACTGTGCC	(GA)_21_	54.5	MT988454
*Aaesti69*	polymorphic	F: [M13]TGAGTGCAACCACTCCAAGT R: CAGCATTACAGAGTCAGTCAATCA	(AT)_21_	54.5	MT988455
*Aaesti71*	polymorphic	F: [M13]TTCCAGACTGCAGAGGTGC R: AATCAAGCATTCGATAGCCG	(AT)_21_	54.5	MT988456
*Aaesti72*	polymorphic	F: [M13]CAAGGTTTAGGAAATTAAGAGCAA R: AATGCATTTCTTTCTGCAGTTT	(AT)_21_	54.5	MT988457
*Aaesti73*	monomorphic	F: [M13]CACTGCATTACAATCCAATAAACA R: AATTCGAATGTTCTTTGACCC	(TA)_21_	50.8	MT988458
*Aaesti75*	polymorphic	F: [M13]CTTTGGTTCAAAGGTGTGGAG R: AAATAGAGGACGGCCCTTAGC	(TA)_21_	54.5	MT988459

Basic summary statistics for each locus (i.e. allele size, number of alleles per locus (Na), number of private alleles, observed (H_O_) and expected (H_E_) heterozygosities, as well as tests for Hardy-Weinberg equilibrium (HWE) were calculated in GenAlEx v. 6.503 [21] based on results obtained for two samples collected from Drawa and Gowienica rivers, respectively. Tests for linkage disequilibrium were performed using Genepop v. 4.7.5 [22, 23]. Micro-Checker v. 2.2.3 software was used to examine large allele dropout, stuttering, and null alleles as potential sources of error [24]. The significant values for all diversity tests of significance were corrected by the sequential Bonferroni’s procedure [25].

## Results and discussion

The advances in high-throughput sequencing technologies enable the rapid and cost-effective development of microsatellite markers for a vast number of different taxa. Currently, Illumina has becomes the first-choice platform used to accomplish microsatellite isolation. In the present study, the *A. aestivalis* whole-genome library was sequenced using the Illumina MiSeq protocol. Obtained paired-end reads were processed and overlapped into 2,378,426 sequences. A total of 234,634 sequences contained at least one microsatellite. Further analyses revealed among them 34,583 unique sequences contained microsatellites and for 15,049 of them, primer designing was possible. Those sequences contained three pentanucleotide motifs, 43 tetranucleotide motifs, 1030 trinucleotide motifs, and 13984 dinucleotide motifs. Mononucleotide repeats were excluded from analyses. Selected 267 sequences containing microsatellite motifs were used for in silico primer designing. Among them, two contained pentanucleotide repeats, 43—tetranucleotide repeats, 56—trinucleotide repeats, and 166—dinucleotide repeats. For the preliminary screening under laboratory conditions, 75 loci out of these 267 potential microsatellite markers were tested in 14 *A. aestivalis* individuals collected from different sampling sites. Primers pairs that failed to amplify target loci, as well as primers that amplified regions of unexpected size, or amplified markers showing stuttering patterns during genotyping, were discarded. A final suite of 56 microsatellite loci yielded consistent amplification (Table 2). Sequences containing these markers were deposited in GenBank under accession numbers MT988404-MT988459.

Polymorphism of 56 selected microsatellite loci was then analyzed among specimens collected from two populations inhabiting unconnected rivers (Drawa and Gowienica, respectively). Analyses revealed 22 monomorphic loci and 34 loci which were polymorphic and consistently amplifiable. Further analyses of these informative loci showed that the total number of observed alleles per locus ranged from 2 to 21 in separated samples, and from 2 to 22 in the overall sample (Table 3). Private alleles were noticed in all but four loci (not observed at loci *Aaesti*12, *Aaesti*15, *Aaesti*19, and *Aaesti*22). Observed heterozygosity ranged from 0.000 (at loci *Aaesti*5 and *Aaesti*21 only two different homozygotes were observed per each locus among individuals from the Drawa river and similarly, two different homozygotes were noticed at locus *Aaesti*21 among individuals from Gowienica river) to 0.933 across loci. In turn, expected heterozygosity ranged from 0.064 (*Aaesti*5 in DR sample and *Aaesti*21 in both DR and GOW samples) to 0.925 (*Aaesti*39 in GOW sample). Within the overall sample, the same parameters ranged from 0.000 to 0.898 and from 0.065 to 0.935, respectively. Null alleles were identified in 26 microsatellite loci and that in turn may resulted in a heterozygote deficiency (Table 3) [26]. The test of linkage disequilibrium showed a non-random association among alleles at 7 pairs of identified loci (i.e. *Aaesti*5-*Aaesti*28, *Aaesti*8-*Aaesti*16, *Aaesti*8-*Aaesti*22, *Aaesti*-16-*Aaesti*69, *Aaesti*8-*Aaesti*72, *Aaesti*19-*Aaesti*26, and *Aaesti*59-*Aaesti*72). This may be a result of physical linkage (loci may be located on the same chromosome, perhaps in close proximity to each other) or other phenomena (e.g. drift, selection, and/or gene flow). Consequently, one of the two associated markers should be excluded from further population genetic analyses in order to avoid the overstating of population structure [27].

**Table 3:** Characterization of the 34 polymorphic microsatellite loci screened in 60 *Aphelocheirus aestivalis* individuals collected from Drawa and Gowienica rivers.

Locus	Allele size (bp)	Na			Private alleles		H_O_			H_E_			HWE-*P*/significance			Null alleles		
		DR	GOW	Overall	DR	GOW	DR	GOW	Overall	DR	GOW	Overall	DR	GOW	Overall	DR	GOW	Overall
*Aaesti5*	116-128	2	3	3	–	1	0.000	0.033	0.017	0.064	0.326	0.496	0.000***	0.000***	0.003**	0.0605	0.2208	0.3203
*Aaesti8*	115-131	3	5	5	–	2	0.172	0.300	0.237	0.372	0.602	0.570	0.000***	0.000***	0.002**	0.1452	0.1886	0.2121
*Aaesti12*	222-230	3	3	3	–	–	0.300	0.517	0.407	0.316	0.574	0.520	0.227	0.119	0.010*	–	–	–
*Aaesti15*	115-139	4	4	4	–	–	0.200	0.310	0.254	0.571	0.571	0.622	0.000*	0.004	0.003**	0.2362	0.1661	0.2266
*Aaesti16*	178-196	4	4	5	1	1	0.250	0.143	0.196	0.631	0.257	0.678	0.000***	0.000***	0.002**	0.2335	0.0908	0.2868
*Aaesti19*	171-177	3	3	3	–	–	0.400	0.333	0.367	0.598	0.523	0.565	0.112	0.035	0.007	0.1238	0.1244	0.1270
*Aaesti21*	113-128	2	2	3	1	1	0.000	0.000	0.000	0.064	0.064	0.065	0.000***	0.000***	0.002**	0.0605	0.0605	0.0610
*Aaesti22*	168-171	2	2	2	–	–	0.233	0.107	0.172	0.375	0.316	0.348	0.039	0.000***	0.006**	–	0.1585	0.1301
*Aaesti26*	147-153	2	3	3	–	1	0.133	0.167	0.150	0.124	0.155	0.140	0.696	0.970	0.05	–	–	–
*Aaesti28*	89-95	4	3	4	1	–	0.467	0.533	0.500	0.574	0.496	0.576	0.322	0.609	0.013*	–	–	–
*Aaesti37*	189-223	14	14	16	2	2	0.821	0.571	0.696	0.893	0.895	0.905	0.247	0.000***	0.003**	–	0.1709	0.1097
*Aaesti38*	190-238	16	21	22	1	6	0.733	0.867	0.800	0.914	0.919	0.928	0.063	0.784	0.005**	0.0946	–	0.0666
*Aaesti39*	100-158	17	19	21	2	4	0.759	0.750	0.754	0.920	0.925	0.935	0.059	0.005	0.003**	0.0842	0.0908	0.0932
*Aaesti40*	104-134	12	11	14	3	2	0.786	0.828	0.807	0.876	0.806	0.852	0.008	0.139	0.003**	–	–	–
*Aaesti41*	114-146	14	9	15	6	1	0.367	0.300	0.333	0.805	0.682	0.757	0.000***	0.000***	0.002**	0.2428	0.2270	0.2413
*Aaesti47*	86-136	15	16	20	4	5	0.417	0.241	0.321	0.912	0.904	0.934	0.000***	0.000***	0.002**	0.2592	0.3481	0.3169
*Aaesti48*	112-142	11	12	15	3	4	0.267	0.700	0.483	0.852	0.854	0.864	0.000***	0.093	0.002**	0.3161	0.0833	0.2044
*Aaesti49*	137-185	13	14	15	1	2	0.310	0.433	0.373	0.870	0.917	0.912	0.000***	0.000***	0.002**	0.2992	0.2524	0.2820
*Aaesti50*	86-114	13	13	14	1	1	0.300	0.310	0.305	0.874	0.871	0.889	0.000***	0.000***	0.002**	0.3065	0.2997	0.3093
*Aaesti51*	92-152	13	16	18	2	5	0.400	0.600	0.500	0.882	0.922	0.917	0.000***	0.000***	0.002**	0.2562	0.1676	0.2173
*Aaesti54*	124-162	14	15	19	4	5	0.500	0.690	0.600	0.834	0.901	0.900	0.000***	0.086	0.005**	0.1819	0.1113	0.1578
*Aaesti55*	143-199	19	17	20	3	2	0.808	0.862	0.836	0.909	0.901	0.916	0.368	0.132	0.017*	–	–	–
*Aaesti56*	88-142	18	16	21	5	3	0.759	0.724	0.741	0.910	0.897	0.928	0.018	0.010	0.004**	0.0794	0.0909	0.0970
*Aaesti59*	118-168	16	15	22	7	6	0.500	0.414	0.456	0.908	0.883	0.909	0.000***	0.000***	0.002**	0.2136	0.2494	0.2372
*Aaesti62*	105-157	18	17	22	5	4	0.700	0.517	0.610	0.909	0.919	0.935	0.084	0.000*	0.003**	0.1094	0.2092	0.1678
*Aaesti63*	137-185	13	13	15	2	2	0.621	0.867	0.746	0.881	0.886	0.908	0.000***	0.486	0.004**	0.1384	–	0.0849
*Aaesti64*	125-165	14	15	17	2	3	0.933	0.862	0.898	0.891	0.880	0.901	0.404	0.778	0.008**	–	–	–
*Aaesti65*	102-120	2	6	6	–	4	0.111	0.467	0.298	0.168	0.544	0.391	0.078	0.000***	0.002**	–	–	–
*Aaesti66*	97-141	15	19	20	1	5	0.931	0.862	0.897	0.897	0.920	0.918	0.111	0.273	0.025*	–	–	–
*Aaesti67*	165-219	17	19	22	3	5	0.733	0.667	0.700	0.896	0.931	0.933	0.016	0.003	0.004**	0.0856	0.1367	0.1204
*Aaesti69*	112-224	13	13	20	7	7	0.207	0.448	0.328	0.734	0.781	0.772	0.000***	0.000***	0.002**	0.3038	0.1869	0.2509
*Aaesti71*	161-193	13	17	17	–	4	0.741	0.897	0.821	0.906	0.912	0.916	0.018	0.108	0.006**	0.0867	–	0.0494
*Aaesti72*	123-161	10	7	14	7	4	0.172	0.138	0.155	0.489	0.399	0.449	0.000***	0.000***	0.001**	0.2125	0.1866	0.2030
*Aaesti75*	123-169	13	18	19	1	6	0.433	0.655	0.542	0.845	0.923	0.902	0.000***	0.000***	0.002**	0.2231	0.1394	0.1893

*DR* drawa river, *GOW* gowienica river, *Na* number of alleles, *H*_*O*_ observed heterozygosity, *H*_*E*_ expected heterozygosity Loci with significant HWE deviations after Bonferroni correction are labeled with an asterisks

*p < 0.05

**p < 0.01

***p < 0.001

The departures out of Hardy-Weinberg equilibrium after Bonferroni correction were found in all but thirteen microsatellite loci. These deviations were mainly due to a deficit of heterozygotes. Low heterozygosity estimated for most identified polymorphic microsatellite markers may be the effect of small size of selected *A. aestivalis* populations, inbreeding and minimal or null immigration of new individuals into populations. On the other hand, observed heterozygosities calculated for some loci (e.g. *Aaesti*40, *Aaesti*55, and *Aaesti*64) are relatively high, indicating that tested *A. aestivalis* populations are not devoid of the intra-population genetic variation. In turn, analyses for connected samples revealed the departures out of Hardy-Weinberg equilibrium after Bonferroni correction only in two identified loci.

Microsatellite markers have proven to be crucial in understanding population genetics and ecology of many species, including freshwater insects (e.g. [28]). Within this group, *A. aestivalis* has a potential as a model organism for the study on the impact of past demographic phenomena, phylogeographic relationships, and infection with endosymbiotic *Wolbachia* on the genetic diversity of slowly dispersing freshwater insects. In the present study, a set of 56 microsatellite markers have been identified for freshwater *A. aestivalis* and 34 of them were polymorphic. These markers provide new suitable tools available for the scientific community to study *A. aestivalis* population dynamics. Finally, the assessment of its genetic diversity and population structure will provide important data, that can be used in population management and conservation efforts, elucidating the broad- and fine-scale population genetic structure of *A. aestivalis*.

## Data Availability

Sequences containing developed microsatellite markers were deposited in GenBank under accession numbers MT988404-MT988459.
